# IL-7–mediated expansion of autologous lymphocytes increases CD8^+^ VLA-4 expression and accumulation in glioblastoma models

**DOI:** 10.1172/JCI181471

**Published:** 2025-04-17

**Authors:** Kirit Singh, Kelly M. Hotchkiss, Sarah L. Cook, Pamy Noldner, Ying Zhou, Eliese M. Moelker, Chelsea O. Railton, Emily E. Blandford, Bhairavy J. Puviindran, Shannon E. Wallace, Pamela K. Norberg, Gary E. Archer, Beth H. Shaz, Katayoun Ayasoufi, John H. Sampson, Mustafa Khasraw, Peter E. Fecci

**Affiliations:** 1 The Preston Robert Tisch Brain Tumor Center,; 2 Department of Neurosurgery, and; 3 The Marcus Center for Cellular Cures, Duke University Medical Center, Durham, North Carolina, USA.; 4 University of Colorado School of Medicine, Anschutz Medical Campus, Aurora, Colorado, USA.; 5 Duke Center for Brain and Spine Metastasis, Duke University School of Medicine, Durham, North Carolina, USA.

**Keywords:** Neuroscience, Oncology, Cell migration/adhesion, Immunotherapy, T cells

## Abstract

The efficacy of T cell–activating therapies against glioma is limited by an immunosuppressive tumor microenvironment and tumor-induced T cell sequestration. We investigated whether peripherally infused nonantigen specific autologous lymphocytes could accumulate in intracranial tumors. We observed that nonspecific autologous CD8^+^ ALT cells can indeed accumulate in this context, despite endogenous T cell sequestration in bone marrow. Rates of intratumoral accumulation were markedly increased when expanding lymphocytes with IL-7 compared with IL-2. Pretreatment with IL-7 ALT also enhanced the efficacy of multiple tumor-specific and nontumor-specific T cell–dependent immunotherapies against orthotopic murine and human xenograft gliomas. Mechanistically, we detected increased VLA-4 on mouse and human CD8^+^ T cells following IL-7 expansion, with increased transcription of genes associated with migratory integrin expression (*CD9*). We also observed that IL-7 increases *S1PR1* transcription in human CD8^+^ T cells, which we have shown to be protective against tumor-induced T cell sequestration. These observations demonstrate that expansion with IL-7 enhances the capacity of ALT to accumulate within intracranial tumors and that pretreatment with IL-7 ALT can boost the efficacy of subsequent T cell–activating therapies against glioma. Our findings will inform the development of future clinical trials where ALT pretreatment can be combined with T cell–activating therapies.

## Abstract 2

T cell immunotherapies are limited by few T cells in glioma. Adoptively transferred lymphocytes expanded with IL-7 exhibit increased VLA-4 expression and accumulate in tumors.

## Graphical abstract

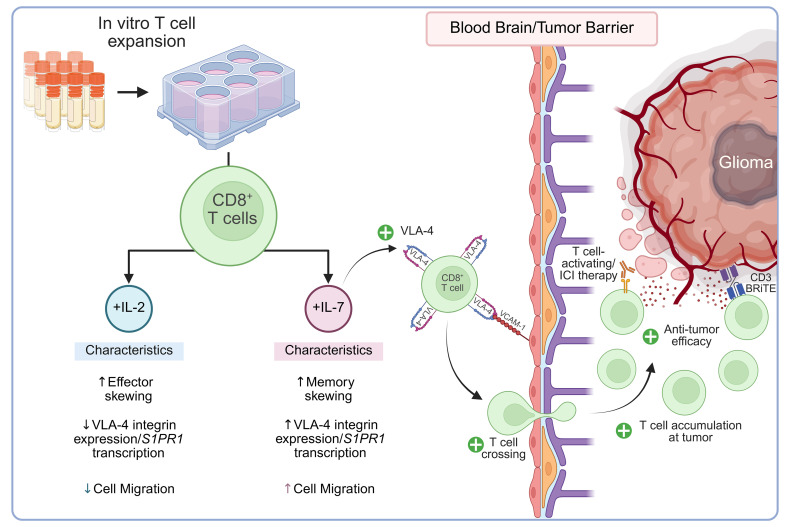

## Introduction

T cell–activating immunotherapies, such as immune checkpoint blockade (ICB), require functional T cells at the tumor site to be effective (1–3). Unfortunately, intracranial tumors are harbored within the immunologically distinct CNS and sit shielded by an immunosuppressive tumor microenvironment (TME) and the blood-brain barrier (BBB) (4). We have shown that CNS tumors, such as glioblastoma, also possess the unique ability to sequester naive T cells within bone marrow (5), limiting their access to the TME. Likewise, we have more recently described a central role for tumor-associated macrophages (TAMs) in driving glioma-infiltrating T cells towards a terminally exhausted state (6), limiting their antitumor capacities. Accordingly, high-grade gliomas possess low numbers of tumor infiltrating lymphocytes (TILs) (7) and have proven minimally responsive to ICB in clinical trials (8–10). This paucity of functional TILs will similarly limit the therapeutic efficacy of newer synthetic therapies that rely on endogenous T cells, including brain bispecific T cell engagers (BRiTEs) (11). Strategies for enhancing functional T cell presence within CNS tumors are needed.

Currently, strategies to increase T cell numbers in tumors include expanding the T cell pool intratumorally; encouraging T cell trafficking from the periphery; or adding T cells systemically. Each of these face major challenges. Expansion of local lymphocytes is limited by subsequent severe exhaustion amid tumor-infiltrating CD8^+^ T cells, making them less proliferative to stimulation (12). Recruitment of peripheral lymphocytes can be boosted by immunostimulatory cytokines, e.g., IL-7 (13) or IL-12 (14), but peripheral cytokine administration can induce profound systemic toxicity (15–17). Local administration of cellular therapies or immunostimulatory cytokines is an alternative but requires an invasive procedure, limiting the potential for repeat administrations (18, 19). Ultimately, peripheral infusion of unmodified or CAR-T cells is an established and easily repeatable method of adding lymphocyte populations to tumors within the CNS (20, 21). Modifying this approach, then, we asked whether peripherally infused nonantigen-specific autologous lymphocytes would accumulate in established CNS tumors and synergize with T cell–activating/ICB therapies.

Currently, cellular therapies such as adoptive lymphocyte transfer (ALT) of autologous T cells typically use cytokines such as IL-2 or IL-7 to support ex vivo expansion (22). IL-2 skews lymphocytes towards a terminally differentiated T effector (T_EFF_) phenotype. While T_EFF_ cells do express migratory integrins, they often have a short half life once removed from culture (23, 24). IL-7, in turn, promotes homeostatic T cell expansion and expands the central memory T cell (T_CM_) pool, leading to greater T cell persistence in vivo (25, 26). Peripheral administration of long-acting IL-7 has been shown to enhance T cell accumulation in murine glioma and boosts the efficacy of T cell engagers against solid tumors (13, 27). We therefore sought to evaluate which growth factor might produce a cellular product with favorable BBB penetrance and enhanced antitumor efficacy in models of established intracranial glioma.

Herein, we report that peripherally administered T cells (ALT) lacking antigen specificity accumulate in established murine intracranial tumors, despite tumor-imposed T cell sequestration in bone marrow. Rates of intratumoral accumulation are markedly increased when T cells are expanded with IL-7 (IL-7 ALT), which also increases the efficacy of accompanying immunotherapeutic platforms. We find that IL-7 increases expression of the migratory integrin very late antigen-4 (VLA-4) on both mouse and human CD8^+^ T cells, while VLA-4 blockade abrogates the enhanced accumulation of IL-7 ALT in tumors. Transcriptional analysis of hPBMCs expanded with IL-7 reveals upregulation of genes involved in integrin expression and tumor infiltration (e.g., *CD9*, *VLA-6*, *EPHA4*). Also upregulated is *S1PR1* (sphingosine-1-phosphate receptor 1, also described as S1P1), increased levels of which are known to avert T cell sequestration and license immunotherapeutic responses to glioma (5). These results provide a route to optimizing ALT expansion, as well as to using ALT as an immunotherapeutic adjunct.

## Results

### IL-7–expanded CD8^+^ T cells demonstrate increased accumulation within orthotopic glioblastoma models despite endogenous T cell sequestration in bone marrow.

Though peripherally administered, activated autologous T cells have been shown to cross the BBB under physiologic conditions (28), their ability to enter the CNS in the setting of tumor-directed T cell sequestration was unclear. Previous work by our group established that sequestration predominantly impacts naive T cells (endogenous or ALT), while memory T cells are largely unperturbed (5). We therefore began by examining the rate of CNS tumor accumulation for ex vivo–expanded nonspecific autologous T cells.

First, we developed T cell expansion processes that skewed phenotypes towards effector (T_EFF,_ CD44^high^CD62L^low^) or central memory (T_CM,_ CD44^high^CD62L^high^) subsets. Expansion of T cells with either 100 IU/mL IL-2 or 20 ng/mL IL-7 was chosen to enrich for CD8^+^ T_EFF_ or CD8^+^ T_CM_ phenotypes, respectively, based on prior experience and published protocols (22, 29, 30). Following expansion, flow cytometry was performed to assess the makeup of the ALT product preadministration (gating and expansion strategy, Supplemental Figure 1, A and B; list of antibodies used for murine and human studies, Supplemental Table 1 and 2; supplemental material available online with this article; https://doi.org/10.1172/JCI181471DS1). Ex vivo expansions with both IL-2 and IL-7 yielded a cellular product of over 95% CD3^+^ cells (Supplemental Figure 1C) which predominantly consisted of CD8^+^ T cells (approximately 80%, Supplemental Figure 1D). The IL-2–ALT product was preferentially skewed towards T_EFF_ (Supplemental Figure 1E) while the IL-7–ALT product skewed towards T_CM_ (Supplemental Figure 1F). Few naive CD8^+^ T cells (T_N_, CD44^low^CD62L^high^) were present at the end of expansion with either cytokine (Supplemental Figure 1G).

To establish the trafficking capabilities of nonspecific CD45.1^+^ T cells in the setting of tumor, we intracranially implanted CT2A stably transfected with the glioma-specific neoantigen Epidermal Growth Factor Receptor variant III (EGFRvIII) into congenic CD45.2^+^ C57/BL6 mice. CT2A is syngeneic on the C57 background and exhibits high heterogeneity, stemness, and resistance to therapy (31). We have also previously described tumor-induced T cell sequestration in bone marrow using this model (5).

**Figure 1:**
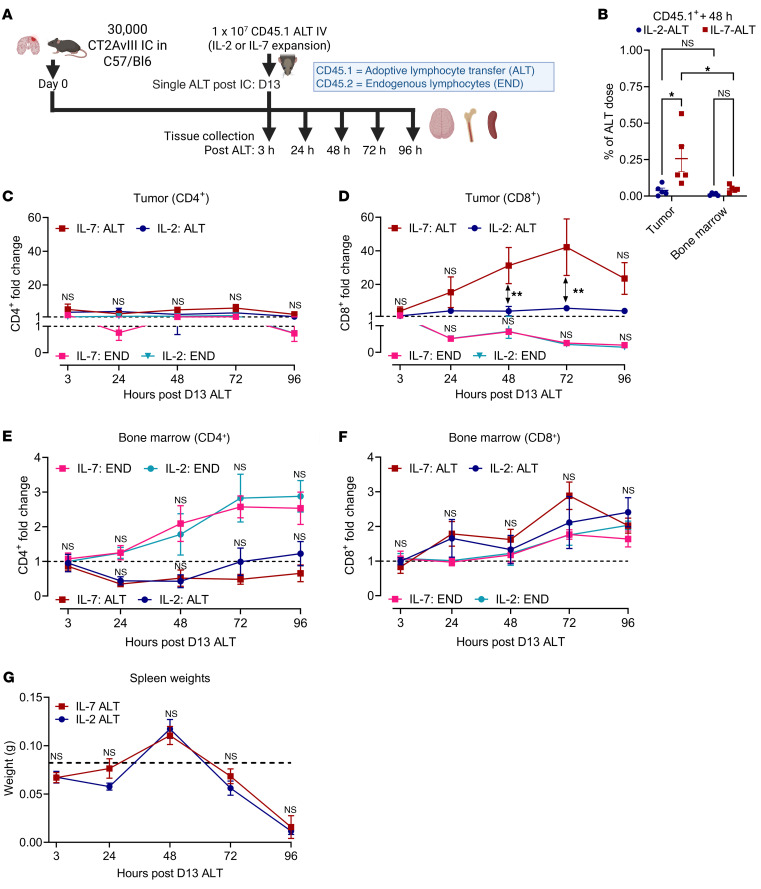
IL-7–ALT CD8^+^ cells demonstrate increased accumulation within orthotopic glioblastoma models despite endogenous T cell sequestration in bone marrow. (**A**) Study overview (*n* = 4–5 mice/group/time point for all graphs). (**B**) Percentage of IL-2–ALT and IL-7–ALT CD45.1^+^ dose detected in tumor by 48-hours after administration (CD4^+^ & CD8^+^ T cells). (**C**) Fold-change relative to Day 0 of exogenous CD45.1^+^CD4^+^ T cell entry and (**D**) CD45.1^+^CD8^+^ T cell entry into brain tumors. (**E**) Fold-change relative to Day 0 of exogenous CD45.1^+^CD4^+^ T cell entry and (**F**) CD45.1^+^CD8^+^ T cell entry into bone marrow. (**G**) Time course of spleen sizes following ALT. Weights from control mice represented by dashed line (0.0823g, *n* = 5). Dashed line in **C**–**F** represents baseline (i.e., 1x). Statistical analyses performed via 2-way ANOVA and data presented as mean ± SEM unless otherwise specified. Experimental outline generated using BioRender.com. *P < 0.05, **P ≤ 0.01.

After tumors were well established at 13 days, mice were given a single ALT of either IL-2– or IL-7–expanded CD45.1^+^ T cells (Figure 1A). Following ALT, tumor and bone marrow were collected daily from separate mice over 4 days. Flow cytometry was used to measure changes to the viable exogenous (CD45.1^+^) and endogenous (CD45.2^+^) CD3^+^ T cell compartment (flow gating in Supplemental Figure 2A). By 48 hours after administration, we detected an increased proportion of the IL-7–ALT dose within tumor compared to IL-2–ALT. (Figure 1B, IL-7 ALT versus IL-2 ALT in tumor, *P < 0.05). No significant difference between dose proportions in bone marrow was seen at the same timepoint (Figure 1B, *P* = 0.8172). Examination of intratumoral T cells over time found that accumulation rates for IL-7–ALT CD8^+^ but not CD4^+^ lymphocytes were significantly greater than for IL-2–ALT. In both groups, endogenous CD8^+^ T cell populations in tumor declined (time course shown for CD4^+^ T cells in Figure 1C, CD8^+^ T cells in Figure 1D. 48-hour IL-7–ALT CD8^+^ T cells 31.24× fold-increase versus IL-2–ALT 4.14 × **P < 0.01, 72-hour IL-7–ALT CD8^+^ T cells 42.2 × versus IL-2–ALT 5.88 ×, **P < 0.01). Counts of IL-7–ALT CD8^+^ T cells in tumor were also increased compared with IL-2 ALT throughout, though differences were nonsignificant (Supplemental Figure 2B).

Simultaneously, increases in endogenous CD4^+^ and CD8^+^ T cell populations in bone marrow were seen across groups over time (CD4^+^ T cells in Figure 1E, CD8^+^ T cells in Figure 1F). Greater fold-change increases in endogenous CD4^+^ T cell accumulation in bone marrow were observed compared with CD8^+^ T cells, in keeping with previous descriptions of tumor-induced CD4^+^ T cell sequestration (endogenous CD4^+^ fold-change of 2.69× versus CD8^+^ T cells fold change of 1.76× at 72-hour observation point) (5, 32, 33). ALT entry into bone marrow was also observed, although this was restricted to the CD8^+^ population. Rates of ALT accumulation in bone marrow did not differ between expansion conditions (Figure 1F, nonadjusted IL-7–ALT CD8^+^ T cell counts in Supplemental Figure 2C). Increases in spleen weights were also seen following ALT, before a subsequent precipitous decline below baseline (time course in Figure 1G). We concluded then that nonantigen specific, exogenously administered CD8^+^ T cells can indeed accumulate within gliomas, despite tumor-imposed T cell sequestration, with IL-7 ALT accumulating intratumorally at a greater rate than IL-2–ALT.

### IL-7 ALT synergizes with T cell–centric immunotherapies in orthotopic glioma models.

**Figure 2:**
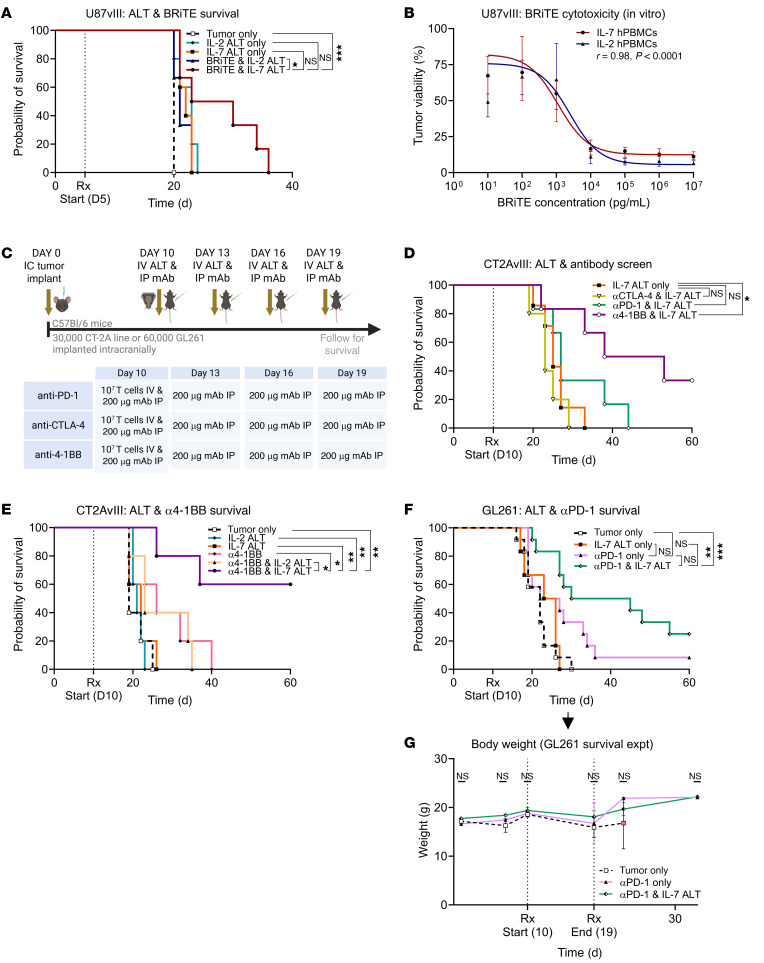
IL-7 ALT synergizes with specific and nonspecific T cell–activating/checkpoint blockade therapies in orthotopic glioma models. (**A**) Evaluation of IL-7 and IL-2 ALT combined with hCD3:EGFRvIII BRiTE in NSG mice. Mice (*n* = 5–6 /group) were implanted with U87vIII and treated with 5 × 10^6^ IV hPBMCs on day 5, with serial IV BRiTE (50 μg) on days 5–9. (**B**) Cytotoxicity assay using BRiTE cocultured with tumor cells (U87vIII) and hPBMCs expanded with IL-2 or IL-7. Nonlinear fitted dose-response curves shown with SEM (*n* = 10–12/group, Pearson Correlation Coefficient *r* = 0.98, ****P < 0.0001). (**C** and **D**) Study overview and findings of a screening approach to identify the best combinatorial mAb approach with ALT (*n* = 5–7/group). (**E**) Survival of combination α4-1BB & IL-7 ALT therapy compared to monotherapy controls treated using same regimen in (**C**) (*n* = 5/group). (**F**) C57BL/6J mice implanted with 6 × 10^4^ GL261 and treated using same regimen in (**C**) with αPD-1 (*n* = 6–12/group, pooled data across 2 experiments). (**G**) Monitoring of bodyweight throughout the treatment period and for two weeks after for survival experiment in **F** (*n* = 6–7/group). Comparison via multiple unpaired *t* tests and data presented as mean ± SEM. Survival comparisons performed via a log-rank (Mantel-Cox) χ^2^ test. Experimental outlines generated using BioRender.com. *P < 0.05, **P ≤ 0.01, ***P < 0.001, ****P < 0.0001.

We next examined whether IL-2 or IL-7 ALT would each enhance survival when combined with T cell–activating immunotherapies. In a first set of experiments, we combined each ALT with EGFRvIII-BRiTE in a U87 xenograft model stably transfected with EGFRvIII (U87vIII). EGFRvIII-BRiTE is a bispecific T cell engager possessing specificity for both the CD3 receptor and the glioma-specific antigen EGFRvIII (34). As EGFRvIII-BRiTE is fully humanized, we evaluated efficacy in a NOD scid gamma (NSG) mouse model reconstituted with either IL-7– or IL-2–expanded human (h) PBMCs. We observed that IL-7 ALT yielded a significant but modest survival benefit when combined with BRiTE, compared with the IL-2–ALT-BRITE combination (Figure 2A, *P < 0.05). Interestingly, no differences between the two modalities were observed in vitro (Figure 2B).

Having observed a survival benefit with the combination of IL-7 ALT + BRITE, we looked to see how successfully IL-7 ALT might also combine with various checkpoint or T cell–activity modifying therapies that typically have limited or no success in CT2A models (outline in Figure 2C). After CT2AvIII tumors were established for 10 days, animals were treated with IL-7 ALT alone, or in combination with α (anti). CTLA-4 (antagonist), αPD-1 (antagonist) or α4-1BB (agonist) monoclonal antibodies. We observed a small trend toward prolonged survival when combining IL-7 ALT with αPD-1 therapy (2 of 6 mice surviving more than 30 days, 0 of 6 with long-term overall survival), but a significant increase in survival when combining IL-7 ALT + α4-1BB compared with IL-7 ALT only (Figure 2D, *P < 0.05). We therefore selected the IL-7 ALT + α4-1BB combination approach for further experiments in this model and performed controlled survival studies using the same timing schematic as above (Figure 2E). We again found that IL-7 ALT + α4-1BB therapy yielded a significant survival benefit, this time compared to combination IL-2–ALT and α4-1BB (*P < 0.05); α4-1BB alone (*P < 0.05); either IL-2 or IL-7 ALT only (both **P < 0.01); or to untreated controls (**P < 0.01). Interestingly, while combination IL-7 ALT + αPD-1 therapy exhibited a limited survival benefit in the CT2AvIII glioma model (Figure 2D), greater efficacy was observed in mice bearing more immunogenic GL261 gliomas (35) (Figure 2F).

We likewise evaluated whether adding IL-7 ALT to clinically relevant checkpoint blockade therapy would result in toxicity. Body weights during and for 2 weeks following treatment were recorded in the GL261-bearing mice whose survival is depicted in Figure 2F. No differences between groups were seen (Figure 2G). To evaluate toxicity in greater detail, we performed acute toxicity studies in healthy mice receiving IL-7 + αPD-1 therapy, comparing against sham controls (outline shown in Supplemental Figure 3A). Body weights again remained stable throughout (Supplemental Figure 3B). Five days after the end of treatment, blood, liver tissue, spinal cord, and brain were taken to assess for liver function, changes in cellular architecture, and potential demyelination. No significant difference in serum chemistry was seen between groups (Supplemental Figure 3C). Review of brain and spinal cord by a board-certified veterinary pathologist also found no differences in terms of histological features, cellular architecture, or demyelination (representative photomicrographs in Supplemental Figure 3, D and E). Regarding liver, no histopathologic abnormalities related to lymphocytic infiltration were noted in the sham group, though presumed perimortem acute hepatocellular apoptosis/necrosis without associated inflammation was seen in 2 of 5 animals. In the combination group, pathologist review identified small numbers of randomly distributed foci (1 to 4 per animal) of acute to subacute hepatocellular apoptosis/necrosis associated with a mixed lymphocytic infiltrate (representative photomicrographs in Supplemental Figure 3F). While these findings were considered to be treatment-related effects, they were considered unlikely to indicate systemic toxicity given the unchanged liver function tests.

### IL-7–expanded CD8^+^ T cells accumulating in tumors exhibit both central and effector memory phenotypes.

Frequent antigenic stimulation upregulates trafficking molecules on T_CM_ cells (36, 37), while T_EFF_ cells also upregulate migratory ligands such as LFA-1 and VLA-4, enhancing endothelial binding following activation (23, 24). We therefore evaluated the phenotypic makeup of exogenous T cells that entered the brain following ALT, as well as their expression of migratory integrins.

**Figure 3:**
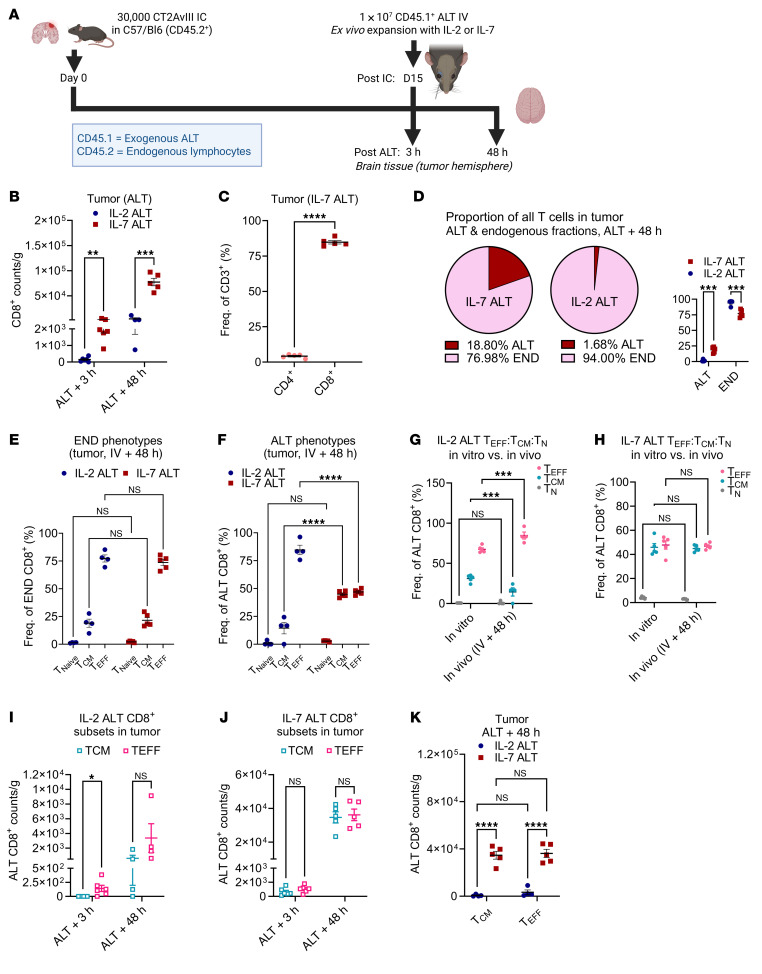
IL-7–expanded CD8^+^ T cells accumulating in tumor consist of both central and effector memory phenotypes. (**A**) Study overview in C57BL/6J mice (*n* = 4–6/group for all graphs). (**B**) Weight-adjusted counts of CD45.1^+^CD8^+^ T cells 3- and 48-hours following ALT in tumors (multiple unpaired *t* tests shown). (**C**) Proportion of IL-7–ALT cells in tumor that are CD4^+^ or CD8^+^ T cells (unpaired *t* test). (**D**) Proportions of exogenous ALT to endogenous T cells in tumor 48 hours following administration, with paired dot-plot showing individual values across groups. (**E**) Comparison of Endogenous (CD45.2^+^) or exogenous/ALT (CD45.1^+^) (**F**) CD8^+^ T_N_, T_CM_, T_EFF_ proportions between IL-2 and IL-7 ALT in tumor 48 hours following administration. (**G** and **H**) Comparison of IL-2 and IL-7 ALT phenotype fractions in tumor to ALT preadministration. (**I** and **J**) Weight normalized counts at both 3- and 48-hour timepoints of CD8^+^ T_CM_ and CD8^+^ T_EFF_ accumulation in tumor when treating with IL-2 or IL-7 ALT (multiple unpaired *t* tests shown). (**K**) Comparison of CD8^+^ T_CM_ and CD8^+^ T_EFF_ presence in tumor following IL-2–ALT or IL-7 ALT. Statistical analyses performed via 2-way ANOVA and data presented as mean ± SEM unless otherwise specified. Experimental outline generated using BioRender.com. *P < 0.05, **P ≤ 0.01, ***P < 0.001, ****P < 0.0001.

To begin, we intravenously administered CD45.1^+^ IL-2 ALT or IL-7 ALT to CD45.2^+^ mice with established (15-day) CT2AvIII tumors. Tumors were collected 3- and 48-hours after ALT (outline in Figure 3A). Again, we observed significantly enhanced accumulation of IL-7–ALT CD8^+^ T cells compared with IL-2–ALT cells (Figure 3B, 3-hours **P < 0.01, 48-hours ***P < 0.001). Similar to previous findings, ALT in tumors primarily consisted of CD8^+^ T cells (Figure 3C, CD8^+^ versus CD4^+^ T cells, ****P < 0.0001). By the 48-hour timepoint, IL-7–ALT T cells made up approximately 20% of all T cells in tumor, (Figure 3D, mean 18.8%, SD ±4.3%, *n* = 5), while the fraction of T cells that consisted of IL-2 ALT in tumors was approximately 1% (mean 1.7%, SD ±1.5%, *n* = 4).

Evaluating the phenotypic composition of the CD8^+^ T cells infiltrating tumor, we observed that at 48-hours after ALT administration, the endogenous CD8^+^ T cell phenotypes in tumors were similar, regardless of whether ALT was expanded with IL-2 or IL-7. Phenotypes consisted mainly of T_EFF_ cells (Figure 3E). Regarding administered (exogenous) ALT CD8^+^ T cells, IL-2 ALT in tumor also predominantly consisted of T_EFF_ cells, while IL-7 ALT consisted of a mixed central/effector (T_CM_/T_EFF_) population (Figure 3F). Naive populations were negligible and did not vary between groups. Given this, we considered that enhanced entry or retention might be more characteristic of the T_CM_ population. However, we noted that the makeup of ALT in tumors was similar to the phenotypic makeup of the IL-2– and IL-7–ALT cell product preadministration (IL-2 ALT in vitro (preadministration) versus in vivo (postadministration) in Figure 3G; IL-7 ALT in Figure 3H). This led us to consider instead whether the increased fraction of CD8^+^ T_CM_ in tumor for the IL-7–ALT group simply reflected the composition of the preadministration product.

To clarify, we compared accumulation of the CD8^+^ T_CM_ and T_EFF_ subsets between both ALT groups. Both the IL-7–ALT CD8^+^ T_EFF_ and T_CM_ subsets were present in tumor in significantly greater numbers than their IL-2–ALT counterparts (individual subsets for each ALT condition in Figures 3, I and J, comparison between ALT conditions in Figure 3K, IL-7–ALT versus IL-2–ALT T_CM_ and T_EFF_ ****P < 0.0001). We concluded that enhanced accumulation of IL-7–ALT CD8^+^ T cells in tumors was more a feature of IL-7 expansion and not reflective of T_CM_ phenotype.

### Expansion with IL-7 upregulates expression of the promigratory integrin VLA-4 on murine CD8^+^ T cells, which is required for enhanced intratumoral accumulation.

**Figure 4:**
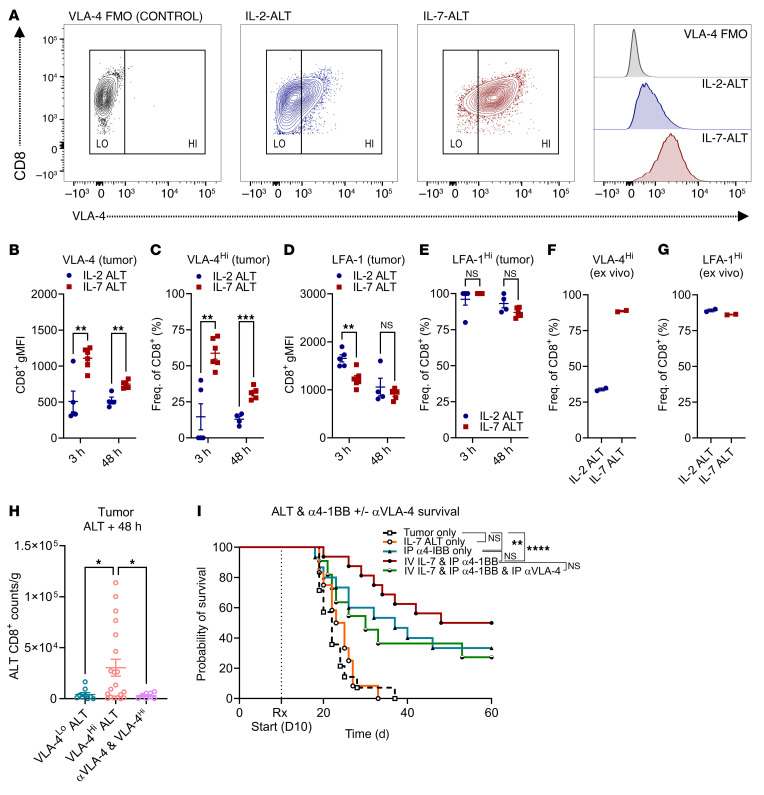
Expansion with IL-7 upregulates expression of the promigratory integrin VLA-4 on murine CD8^+^ T cells, which is required for enhanced intratumoral accumulation. (**A**) Representative gating strategy shown for VLA-4 expression on CD8^+^ T cells from IL-2 or IL-7 ALT. (**B** and **C**) CD8^+^ T cell VLA-4 expression (gMFI) and proportion of CD8^+^ VLA-4^Hi^ T cells shown when expanding with IL-2 or IL-7 ALT (experiment outline in Figure 3, n = 4–6/group). (**D** and **E**) CD8^+^ T cell LFA-1 expression (gMFI) and proportion of CD8^+^ LFA-1^Hi^ T cells shown when expanding with IL-2 or IL-7 ALT. (**F** and **G**) Analysis of the CD8^+^ T cell VLA-4^Hi^ or LFA-1^Hi^ proportion in ALT cellular product at expansion end (2 technical replicates). (**H**) Entry of CD45.1^+^CD8^+^ T cells in tumor following VLA-4^Lo^, VLA-4^Hi^ ALT or VLA-4^Hi^ ALT, and αVLA-4 (single-dose 200 μg intraperitoneal αVLA-4 antibody (BioXCell) pre-ALT, 3 pooled experiments shown, *n* = 8–20/group, 1-way ANOVA shown). (**I**) Evaluation of VLA-4 expression on the endogenous CD8^+^ T cell compartment over time (IL-2 and IL-7 treatment groups pooled, *n* = 9–12/group). Survival comparisons performed via log-rank (Mantel-Cox) χ^2^ test. Statistical analyses performed using unpaired *t* tests and data presented as mean ± SEM unless otherwise specified. *P < 0.05, **P ≤ 0.01, ***P < 0.001.

To better understand the impact of IL-7 on T cell accumulation in tumors, we analyzed the expression of migratory integrins common to all lymphocytes (VLA-4, LFA-1) on exogenous T cells that entered the CNS using flow cytometry (gating strategy in Figure 4A). We observed that both the expression of the migratory integrin VLA-4 and the fraction of VLA-4^Hi^ cells was significantly increased amid IL-7–ALT CD8^+^ T cells arriving early in tumor compared with IL-2 ALT, though this difference subsided by the 48 hour sampling point (gMFI in Figure 4B, 3 hours and 48-hours **P < 0.01, CD8^+^ T cell VLA-4^Hi^ % in Figure 4C). Conversely, we saw increased LFA-1 expression on IL-2–ALT CD8^+^ T cells compared with IL-7 ALT at the first sampling timepoint (Figure 4D, **P < 0.01 LFA-1 gating in Supplemental Figure 4A). The percentage of CD8^+^ LFA-1^Hi^ T cells was similarly high throughout for both conditions (Figure 4E). The preadministration fraction of CD8^+^ VLA-4^Hi^ T cells was also higher amid the IL-7 ALT, while the fraction of preadministration CD8^+^ LFA-1^Hi^ T cells was similar between groups (CD8^+^ VLA-4^Hi^ T cells in Figure 4F, CD8^+^ LFA-1^Hi^ T cells in Figure 4G, technical repeats for each ALT condition shown).

To evaluate the impact of VLA-4 levels on T cell trafficking to tumor, we again used IL-7 to generate an ALT consisting of CD4^+^ and CD8^+^ T cells that had increased VLA-4 expression compared with baseline levels seen when expanding with IL-2 (VLA-4^Hi^ or VLA-4^Lo^ ALT, pooled ALT expression shown in Supplemental Figure 4B). VLA-4^Hi^ or VLA-4^Lo^ ALT were then separately administered to animals with 15-day established CT2AvIII tumors, either alone or alongside a VLA-4 blocking antibody (BioXCell, 200 mg intraperitoneally (IP)). VLA-4^Hi^ ALT CD8^+^ T cells demonstrated significantly enhanced accumulation within tumors compared with VLA-4^Lo^ ALT, accumulation that was abrogated by coadministration of αVLA-4 (VLA-4^Hi^ versus VLA-4^Lo^, *P < 0.05; VLA-4^Hi^ versus αVLA-4 & VLA-4^Hi^, *P < 0.05, 3 pooled experiments shown, Figure 4H). Administration of αVLA-4 was also found to reduce the therapeutic efficacy of combination IL-7 ALT + α4-1BB seen in the CT2AvIII model. Here, we observed that IP αVLA-4 reduced survival in the combination therapy group from 54 to 30 days, similar to monotherapy groups, though these curves were not significantly different (IP α4-1BB only: 37 days, 3 pooled experiments shown, Figure 4I). Based on these findings, we concluded that: (a) IL-7 exposure increases the expression of VLA-4 on CD8^+^ T cells and (b) VLA-4 expression and signaling are necessary for the enhanced accumulation of IL-7 ALT CD8^+^ T cells in glioma.

### Intratumoral lymphocytic VLA-4 and endothelial/pericytic VCAM-1 expression increase over time in glioma.

**Figure 5:**
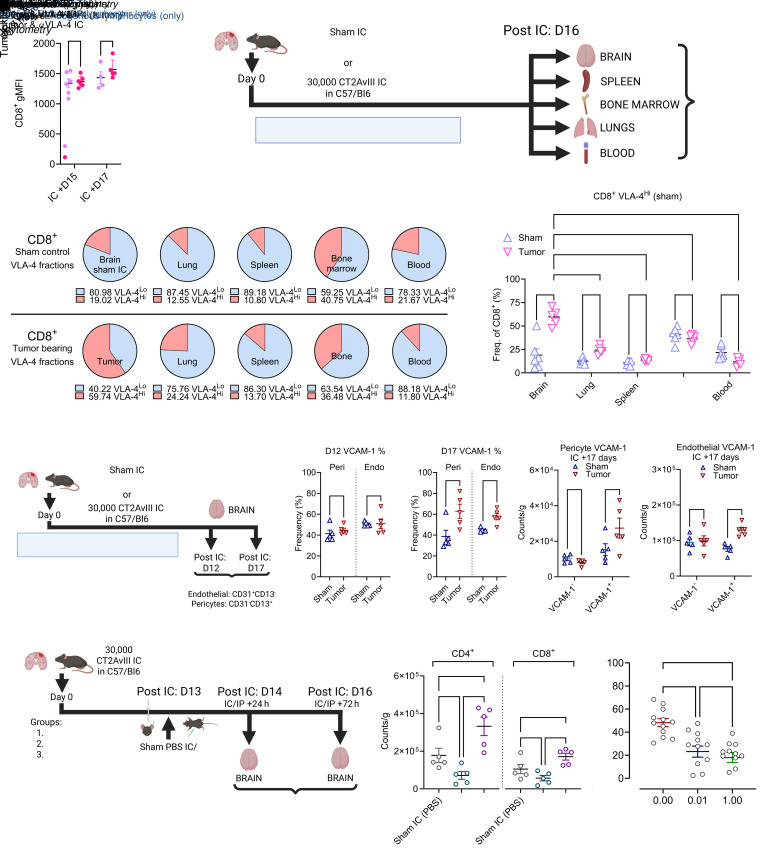
Lymphocytic VLA-4 & endothelial/pericytic VCAM-1 expression increases over time on native T cells in murine glioma. (**A**) Comparison of CD8^+^ T cell VLA-4 gMFI expression on endogenous cells over time from prior experiment (shown in Figures 3 and 4). (*n* = 4–6/group). (**B**) Study overview to assess VLA-4 expression across compartments. (**C** and **D**) Evaluation of VLA-4^Lo^ and VLA-4^Hi^ fractions across different compartments. Comparisons in **D** via 2-way ANOVA (*n* = 5–6/group). (**E**) Study overview to assess endothelial VCAM-1 in the CNS. No ALT was used. Tumor hemispheres were collected, and endothelial cells (CD31^+^CD13^–^)/pericytes (CD31^–^CD13^+^) were analyzed (*n* = 5/group). (**F** and **G**) Frequency of VCAM-1^+^ pericytes/endothelial cells in tumor at D12 and D17 following tumor/sham injection. (**H** and **I**) Counts of VCAM-1^–^/VCAM-1^+^ pericytes/endothelial cells in tumor compared to sham controls at D17 following intracranial injections. (**J**) Study overview to evaluate intraperitoneal/intracranial VLA-4 blockade (*n* = 5/group). (**K**) Intracranial CD4^+^ & CD8^+^ T cell counts 72 hours following administration of intraperitoneal/intracranial αVLA-4 or sham controls. Comparisons via 1-way ANOVA. (**L**) In vitro cytotoxicity with CT2AvIII, T cells, αVLA-4, and BRiTE at EC_50_ concentration of 0.01 μg/mL. *n* = 12/dose level. Comparisons via 1-way ANOVA. Statistical analyses performed using unpaired *t* tests and data presented as mean ± SEM unless otherwise specified. Experimental outlines generated using BioRender.com. *P < 0.05, **P ≤ 0.01, ***P < 0.001, ****P < 0.0001.

While evaluating ALT VLA-4 expression, we noted that VLA-4 levels were consistently elevated over time on endogenous CD8^+^ T cells in tumor (Figure 5A). Noting the role of VLA-4 in guiding T cells towards inflamed tissue (38), we first examined whether VLA-4^Hi^ T cells were unique to the tumor environment. We performed flow cytometry on various tissues (brain, spleen, bone marrow, lungs, and blood) in mice with established CT-2AvIII tumors or sham-injected controls (experimental outline in Figure 5B, gating strategy in Supplemental Figure 5A). Animals did not receive ALT, allowing for evaluation of endogenous T cell VLA-4 expression without external influence. We again observed a significant increase to the VLA-4^Hi^ fraction amid CD8^+^ T cells, as well as to weight-adjusted VLA-4^Hi^ CD8^+^ T cell counts in brain tumor tissue compared with all other compartments, as well as to brain from sham controls (fractions in Figure 5C, comparisons in Figure 5D, ****P < 0.0001, weight adjusted counts vs sham controls in Supplemental Figure 5B, **P < 0.01). VLA-4^Hi^ CD8^+^ T cells in tumor were overwhelmingly T_EFF_ cells (Supplemental Figure 5C). Outside of the tumor, VLA-4^Hi^ CD8^+^ T cells were most commonly found in the bone marrow (Figure 5, C and D).

As glioblastoma is characterized by high levels of microvascular proliferation (39), we next evaluated if endothelial/pericytic VCAM-1 (VLA-4’s ligand) (40) was also increased in tumor. Recent studies of T cell recruitment in other intracranial tumor models (i.e., melanoma) found negligible VCAM-1^+^ endothelial or pericyte populations (41). To determine if VCAM-1^+^ cells were present in the context of glioma, we collected brain tissue from mice with established intracranial CT2AvIII glioma or sham controls. Both endothelial (CD31^+^CD13^–^) and pericyte (CD31^–^CD13^+^) populations were analyzed via flow cytometry (outline in Figure 5E, gating in Supplemental Figure 5D). Here, we observed a similar frequency of expression of VCAM-1 on endothelial cells and pericytes on Day 12 of tumor growth (approximately 45% VCAM-1^+^), with similar levels also seen across tumors and sham controls (Figure 5F). However, as tumors progressed further, increasing proportions and counts of VCAM-1^+^ endothelial cells and pericytes were observed in tumors compared to sham controls (Figure 5G, pericytes *P < 0.05, endothelial cells **P < 0.01, D17 weight adjusted counts in Figure 5, H and I respectively).

Given this, we questioned whether intratumoral T cell accumulation might be enhanced by blocking the VLA-4–VCAM-1 axis and whether results would differ if blockade was administered systemically vs intracranially. To evaluate, we implanted mice with CT2AvIII gliomas, and 13 days later, we administered αVLA-4 either intraperitoneally or intracranially. As free αVLA-4 antibody levels are 100-fold reduced in CSF compared with serum following systemic infusion (42), we selected dose levels of 200 μg αVLA-4 intraperitoneally and 2 μg intracranially to achieve approximately equivalent exposure (experiment outline in Figure 5J). First, we evaluated the impact of both intraperitoneal and intracranial αVLA-4 on intratumoral CD8^+^ T cell VLA-4 expression and found that both treatment routes suppressed VLA-4 levels on T cells, though this was only sustained when administering intraperitoneally (Supplemental Figure 5E). Next, we evaluated intracranial counts of endogenous CD4^+^ and CD8^+^ T cell populations 24- and 72-hours after αVLA-4 administration. Across treatment groups, no differences were seen at the 24-hour measurement point (Supplemental Figure 5F). By 72-hours, however, intraperitoneal αVLA-4 had elicited reduced counts of both CD4^+^ and CD8^+^ T cells in tumors, even compared with nontumor-bearing controls (24 hour intratumoral counts in Supplemental Figure 5F; 72 hour counts in Figure 5K, intraperitoneal αVLA-4 versus sham, CD4^+^ and CD8^+^ T cells, *P < 0.05). Conversely, intracranial αVLA-4 produced increases in accumulation of both populations within tumors by 72 hours after administration, though this was only significant for CD4^+^ T cells compared with sham controls (Figure 5K, intracranial αVLA-4 versus sham, CD4^+^ T cells *P < 0.05, CD8^+^ T cells *P* = 0.068).

We therefore further investigated the therapeutic potential for intracranial VLA-4 blockade. While increasing intratumoral T cell counts remains a central goal, VLA-4 is involved in immune synapse formation and T cell activation (43), making the net impact of its blockade unclear. To evaluate the effects of VLA-4 blockade on T cell activity, we performed in vitro coculture cytotoxicity assays with CT2AvIII, T cells, and EGFRvIII-BRiTE. αVLA-4 antibody was added at escalating amounts to BRiTE at its EC_50_ dose (0.01 μg/mL, dose-finding data in Supplemental Figure 5G). The addition of αVLA-4 indeed inhibited the T cell antitumor cytotoxicity otherwise mediated by BRiTE (Figure 5L). Thus, while intracranial αVLA-4 may enhance intratumoral T cell accumulation, there appears to be a negative impact on antitumor cytotoxicity.

### IL-7 expansion of hPBMCs from both healthy volunteers and patients with glioblastoma upregulates lymphocyte VLA-4 expression.

We therefore returned to further investigate our initial IL-7–ALT method for enhancing T cell numbers in tumor. To determine the clinical translatability of our approach, we sought to determine whether (a) leukapheresis products from patients with glioblastoma would respond/expand to the same extent as those from healthy controls, and (b) similar changes to VLA-4 expression and T cell migratory potential would occur when expanding human T cells in the presence of IL-7.

To permit relevant comparisons, we obtained leukapheresis products from healthy volunteers and patients with glioblastoma who had given written consent to provide PBMCs for the purposes of T cell expansion. An overview of donor demographics and pathology is shown in Table 1. Median ages across groups were similar (controls 54.3 years (range 43.3–55.4) versus glioblastoma 58.7 years (range 37.4–63.3)) with a 2:1 M:F ratio in both groups. Of the glioblastoma samples, 5 of 6 were collected from patients who had previously received dexamethasone.

**Figure 6:**
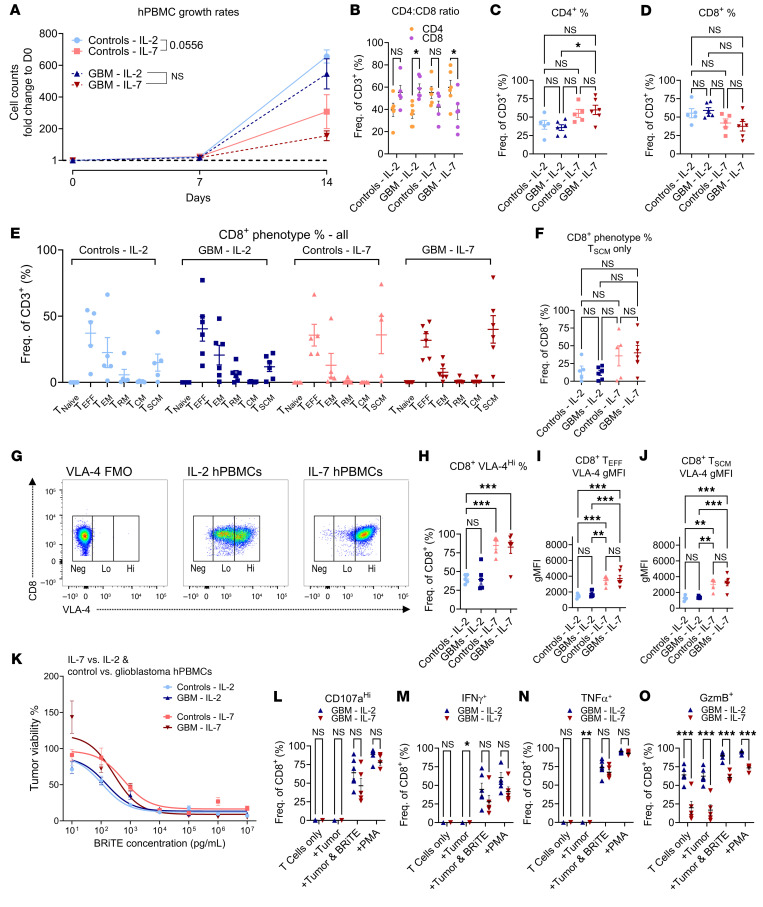
IL-7 expansion of hPBMCs from both healthy volunteers and patients with glioblastoma upregulates lymphocytic VLA-4 expression. (**A**) hPBMC growth rates for IL-2 versus IL-7 coculture from both glioblastoma and control volunteer leukaphereses. (*n* = 5–6/group). (**B**–**D**) CD4^+^:CD8^+^ T cell ratios and comparisons for IL-2 versus IL-7 for control and glioblastoma samples at expansion end. (**C** and **D**) Comparisons in **B** via 2-way ANOVA and, in **C** and **D,** via 1-way ANOVA. (**E**) Overview of CD8^+^ T cell phenotype fractions at expansion end: Naive (T_N_), Effector (T_EFF_), Effector Memory (T_EM_), Resident Memory (T_RM_), Central Memory (T_CM_), Stem Cell Memory (T_SCM_). (**F**) Fraction of CD8^+^ T_SCM_ at expansion end across groups. (**G**–**J**) Gating strategy for VLA-4^Hi^ fraction and proportion of VLA-4^Hi^ in all CD8^+^ T cells (**H**) as well as CD8^+^ T_EFF_ (**I**) and CD8^+^ T_SCM_ cells (**J**) at expansion end. (**K**) Cytotoxicity assay with tumor (U87vIII) cocultured with IL-2 or IL-7 expanded donor/glioblastoma hPBMCs & BRiTE. (**L**–**O**) Degranulation assays assessing CD107^Hi^, IFN-γ^+^, TNF-α^+^, GzmB^+^ in glioblastoma CD8^+^ T cells expanded with IL-2 and IL-7. Comparisons via multiple unpaired *t* tests (*n* = 5–6/group). Statistical analyses performed using 1-way ANOVA and data presented as mean ± SEM unless specified. *P < 0.05, **P ≤ 0.01, ***P < 0.001.

Samples from control and glioblastoma groups were thawed and activated via αCD3/αCD8 stimulation. Cultures were maintained for 14 days in media supplemented with 300 IU/mL IL-2 (based on prior protocols) (44) or 20 ng/mL IL-7. After expansion, cell counts, phenotypes, activation levels, and VLA-4 expression were evaluated (flow gating and schematic in Supplemental Figure 6, A and B). While both IL-7 and IL-2 were capable of independently supporting T cell proliferation (Figure 6A), IL-2 produced greater T cell yields for both control and GBM leukapheresis samples (Supplemental Figure 6C). Perhaps surprisingly, no significant difference between control and glioblastoma T cell expansion rates were observed. The final expanded product consisted of approximately 95% CD3^+^ T cells across all groups (Supplemental Figure 6D). IL-7 expansion skewed significantly more towards CD4^+^ T cells (approximately 60:40 ratio of CD4^+^:CD8^+^ T cells), whereas IL-2 skewed more towards CD8^+^ T cells (CD4^+^:CD8^+^ T cells, approximately 33:66, Figure 6, B–D). On more specific phenotyping of memory and effector subsets, we observed that T_EFF_ (CD45RO^–^CD45RA^+^CD95^+^CCR7^–^) and T Effector Memory (T_EM,_ CD45RO^+^CD45RA^–^CD62L^–^CCR7^–^) fractions were similar across the IL-2 and IL-7 expansion groups (T_EFF_ in Supplemental Figure 6E, T_EM_ in Supplemental Figure 6F, all subsets in Figure 6E). IL-7 did trend toward increasing the stem cell memory fraction (T_SCM,_ CD45RO^–^CD45RA^+^CD95^+^CCR7^+^) compared with IL-2, though changes were nonsignificant (Figure 6F).

We next evaluated whether IL-7 increased VLA-4 expression on human T cells, as we had seen in our mouse models. We observed a biphasic distribution in VLA-4^+^ cells and classed lymphocytes as VLA-4^Neg^, VLA-4^Lo^ or VLA-4^Hi^ (gating in Figure 6G). For both control and glioblastoma samples, IL-7 significantly increased the VLA-4^Hi^ fraction over that seen with IL-2 (Figure 6H, ***P < 0.001, CD4^+^ and CD8^+^ T cell gMFI shown in Supplemental Figure 6, G and H). Significant upregulation of VLA-4 expression was observed on CD8^+^ T_EFF_ and CD8^+^ T_SCM_ subsets from both groups (Figure 6, I and J, **P < 0.01 for healthy controls and ***P < 0.001 for glioblastoma patients). Changes to CD8^+^ T_EM_ VLA-4 expression were nonsignificant (Supplemental Figure 6I).

Finally, we performed in vitro functional assays. These included cytotoxicity assays using our end-expansion product, cultured alongside BRiTE and EGFRvIII^+^ tumor (U87vIII), as described above. Similar dose-dependent tumor killing was observed across all conditions above BRiTE concentrations of 1 × 10^3^ pg/mL (Figure 6K). We then undertook in vitro stimulation assays of sorted CD8^+^ T cells (cytokine gating strategy in Supplemental Figure 7A, sort gating in Supplemental Figure 7B). Sorted CD8^+^ T cells were expanded with IL-2 or IL-7 and cocultured with tumor only (U87vIII); tumor + BRiTE; or phorbol myristate acetate (PMA)/ionomycin. For T cells that were not exposed to either tumor, BRiTE or PMA stimulus were also analyzed to assess background activity. We observed similar results for IL-2– and IL-7–expanded glioblastoma patient CD8^+^ T cells in terms of CD107a^Hi^ degranulation, IFN-γ, and tumor-necrosis factor-alpha (TNF-α) production (Figure 6, L–N). While we did observe increased frequencies of Granzyme B^+^ (GzmB^+^) T cells in the IL-2–expanded group compared with those expanded with IL-7, we also noted an increased baseline frequency of GzmB^+^ T cells in the unstimulated/IL-2–expanded and tumor-only control groups (Figure 6O). Ultimately, then, IL-7 expansion enhanced CD8^+^ T cell VLA-4 expression among hPBMC samples in a manner similar to that seen with murine T cells, while having a similar impact on T cell function to IL-2 exposure.

### IL-7 increases transcription of genes in T cells associated with enhanced migratory function (CD9) and protection against sequestration (S1PR1).

**Figure 7:**
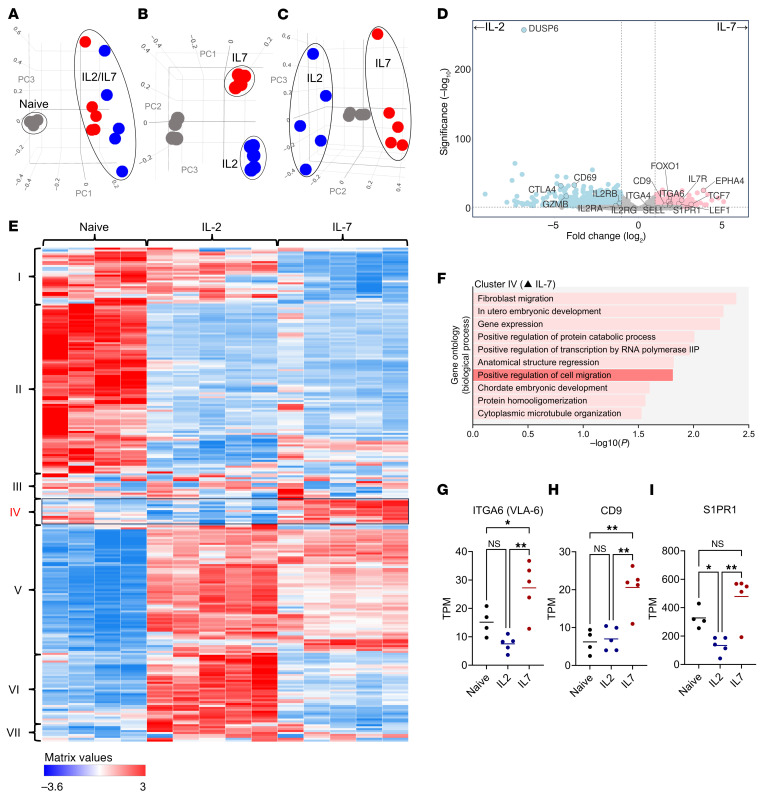
IL-7 upregulates transcription of genes associated with enhanced migratory integrin expression (*CD9*) and protection against tumor-induced sequestration (*S1PR1*). (**A**–**C**) Principal component analysis of naive (gray), IL-2–expanded (blue), and IL-7–expanded (red) CD8^+^ T cells (*n* = 4–5/group for all graphs). (**D**) Volcano plot comparing differential gene expression between IL-2– and IL-7–expanded CD8^+^ T cells. (**E**) Hierarchical cluster heatmaps of the naive, IL-2, and IL-7 groups with seven unique clusters identified (I–VII). (**F**) Gene Ontology biological process analysis of genes expressed in cluster IV (upregulated with IL-7, *EPHA4*, *CD9*, *ITGA6*, *S1PR1*, among others). (**G**–**I**) Comparisons of raw transcripts per million (TPM) across groups for *ITGA6*, *CD9*, and *S1PR1* (comparisons via 1-way ANOVA). PCA analysis was generated using BioJupies (66). Hierarchical cluster heatmaps were generated using clustergrammer (Ma’ayan lab) (67). Gene Ontology (GO) biological process analysis was performed using the NIH DAVID database (53). Volcano plots were generated using VolcaNoseR (69). Comparisons via 1-way ANOVA and data presented as mean ± SEM unless otherwise specified. *P < 0.05, **P ≤ 0.01.

To evaluate IL-7’s effect on migratory integrin expression, we performed bulk RNA sequencing using the same glioblastoma hPBMC samples. CD8^+^ T cells were sorted from naive (preexpansion), IL-2–expanded, or IL-7–expanded products. Principal component analysis revealed that expansion with IL-7 versus IL-2 resulted in distinct CD8^+^ T cell transcriptional fates (Figure 7, A–C). Deeper interrogation of these fates via analysis of differential gene expression revealed that IL-7 significantly increased the transcription of genes involved in T cell migration and trafficking compared with IL-2 (*EPHA4 P*_adj_ = 9.39 × 10^–27^) (45)*, ITGA4* (VLA-4, *P*_adj_ = 0.043) and *ITGA6* (VLA-6, *P*_adj_ = 9.64 × 10^–08^) (46). Genes involved in stem-like/memory T cell development were also significantly increased by IL-7 exposure (*TCF7* which encodes for TCF1, *P*_adj_ = 1.60 × 10^–06^ (47), *FOXO1,* *P*_adj_ = 7.52 × 10^–11^ and *LEF1*, *P*_adj_ = 2.41 × 10^–10^). IL-2–expanded CD8^+^ T cells demonstrated increased transcription of T cell markers associated with effector activity (*CD69*, *P*_adj_ = 2.48 × 10^–^34*, GZMB, P*_adj_ = 2.16 × 10^–18^*)* and exhaustion (*CTLA-4, P*_adj_ = 1.55 × 10^–33^, *TOX P*_adj_ = 0.00068, *HAVCR2* (TIM-3) *P*_adj_ = 0.00018*)*. Volcano plots for all three comparisons are shown in Supplemental Figure 8, A and B (naive versus IL-2 or IL-7, respectively), and Figure 7D (IL-2– versus IL-7–expanded). Analysis of raw transcripts per million (TPM) also found that IL-7 yielded the highest TPM for *ITGA4* (VLA-4), but this was not significantly increased compared with the IL-2 cohort (Supplemental Figure 8C).

Differential expression analysis also revealed a highly significant outlier when expanding with IL-2 vs IL-7. Transcription of Dual-Specificity Phosphatase 6 (*DUSP6*) was increased 6.8× when expanding with IL-2 versus IL-7 (Figure 7D, *P* = 7.85 × 10^–258^). DUSP6 has been shown to negatively regulate the extracellular signal-regulated kinase (ERK1/2) pathway (48–50), which is activated by IL-2 (51). Curious as to the impact of DUSP6, we performed a repeat 2-week expansion where hPBMCs were expanded with IL-2 alone or alongside BCI, a small molecule inhibitor of DUSP6 (52). We found that, while BCI did not increase CD8^+^ T cell VLA-4 expression, it did increase the fraction of CD8^+^ T_SCM_ cells (CD8^+^ T cell VLA-4 gMFI in Supplemental Figure 8D, CD8^+^ T_SCM_ fraction in Supplemental Figure 8E, technical repeats). We surmised that DUSP6 may affect CD8^+^ T cell differentiation but not VLA-4 expression.

Noting that transcription of another migratory integrin (VLA-6) was enhanced with IL-7, we considered whether broader changes in integrin expression might be occurring. We performed hierarchical cluster heatmapping of genes that were differentially expressed across the naive, IL-2, and IL-7 groups. This identified seven clusters (I–VII, Figure 7E). Clusters I and VI were specifically upregulated in the IL-2–expanded samples, while cluster V was upregulated for both. Cluster IV was the only gene set specifically upregulated within IL-7–expanded samples compared with IL-2 expanded and naive CD8^+^ T cells. To interrogate the biological processes that these clusters represented, we performed Gene Ontology (GO) analysis using the NIH DAVID database (53). For clusters I and VI (upregulated in IL-2), we identified biological processes associated with an inflammatory adaptive immune response and positive regulation of T cell activation (Supplemental Figure 8, F and G). These included genes related to cytotoxic T cell activity (*GZMA*, *GZMB*, *IFN-*γ, *FASLG*, *CD69*) as well as markers of T cell exhaustion (*CTLA-4*, *TOX*). For cluster V (upregulated in both), increased transcription of genes associated with cell division were observed (e.g., *MKI67*) (54), (Supplemental Figure 8H).

GO analysis of cluster IV (upregulated in IL-7) identified biological processes associated with positive regulation of cell migration (Figure 7F). These included genes coding for *ITGA6* (VLA-6, TPM comparisons across groups shown in Figure 7G) as well as *CD9*, a protein that has been associated with increased expression of multiple migratory integrins, including VLA-6 and VLA-4 (55, 56). Within our data, *CD9* TPM was significantly and specifically increased within IL-7–expanded hPBMCs compared with naive or IL-2–expanded cohorts (**P < 0.01, Figure 7H). Interestingly, we also identified *S1PR1* (encoding the S1P1 receptor) within cluster IV. Our group has previously reported that loss of S1P1 mediates tumor-induced T cell sequestration in bone marrow (5) and that stabilization of S1P1 on the surface of T cells makes them resistant to sequestration (5). Here, we found that IL-2 significantly reduced *S1PR1* TPM compared with naive controls (*P < 0.05), while IL-7 significantly increased *S1PR1* TPM (***P < 0.001, Figure 7I). We concluded that IL-7 upregulates genes associated with migratory integrin expression, while also increasing *S1PR1* transcription.

## Discussion

For T cell-activating therapies to be effective against tumors of the CNS, they must be able to encounter functional immune cells within the intracranial compartment. Although immune cells can cross the BBB (28), primary CNS malignancies are capable of “driving” T cells away by inducing loss of lymphocytic S1P1, resulting in T cell sequestration within bone marrow (5, 57).

Our results demonstrate that nonspecific CD8^+^ T cells expanded with IL-7 can accumulate in tumor tissue, despite tumor-induced T cell sequestration. Further, we demonstrate that the administration of IL-7 ALT can synergize with multiple T cell–activating therapies against established intracranial glioma. Campian et al. reported a related phenomenon, whereby peripheral administration of long-acting recombinant IL-7 enhanced cytotoxic CD8^+^ T cell numbers systemically and in tumors for similar animal glioma models (13). Another recent study by Lee et al. reports synergism between recombinant IL-7 administration and bispecific T cell engager therapy in solid tumor models (27). Interestingly, they report that IL-7 encourages recruitment of nonexhausted polyclonal bystander CD8^+^ TILs to tumors. Though these bystander TILs do not have direct antitumor activity themselves, they can be redirected by T cell engagers to become tumoricidal. Our study adds to this body of literature, finding that IL-7 enhances expression of migratory integrins like VLA-4, and increases transcription of genes involved in resistance to sequestration (*S1PR1*).

IL-7’s effect on upregulating VLA-4 has been previously reported in vitro (58), and peripheral administration of recombinant human IL-7 (rhIL-7) has been observed to increase VLA-4 and T cell trafficking in murine models of sepsis (59). In our study, RNA sequencing of CD8^+^ T cells from patients with glioblastoma similarly identified that transcription of VLA-4 was greatest when expanding with IL-7. Interestingly, while only IL-7 significantly increased CD8^+^ VLA-4 transcription levels compared with preexpansion lymphocytes, no significant difference in VLA-4 TPM was observed between IL-2– and IL-7–expanded cells. However, evaluation of genes uniquely upregulated by IL-7 did identify transmembrane proteins (*CD9*) associated with multiple VLA integrins (55, 56). In keeping with this finding, we observed significantly increased transcription of *ITGA6* (VLA-6) when expanding with IL-7 compared with IL-2. Though our study found that VLA-4 was necessary for enhanced CD8^+^ T cell accumulation, it is possible that IL-7 increases the expression of multiple proinfiltrative integrins. These could further enhance the ability of CD8^+^ T cells to enter and accumulate in intracranial glioma.

As mentioned, other genes uniquely upregulated by IL-7 included the g-protein–coupled receptor *S1PR1*. We also have previously identified that S1P1 loss mediates T cell sequestration (5). Accordingly, mice with genetically stabilized S1P1 expression in T cells prove sequestration resistant and exhibit superior survival when using immunotherapy in the face of glioma (5). Our findings align with this prior work. Notably, we also identify that IL-2 exposure decreases the transcription of *S1PR1*. This, in turn, may underlie some of the failure of IL-2–expanded cellular products to accumulate in intracranial tumors, though further confirmatory work is required.

We also report here increasing lymphocyte VLA-4 expression and increasing numbers of VCAM-1^+^ endothelial cells/pericytes in the context of glioma over the course of tumor progression. Given that recent studies have reported low fractions of VCAM-1^+^ cells in other intracranial malignancies (e.g., melanoma) (41), this finding suggests that T cell recruitment and BBB trafficking dynamics may vary between cancer types. To identify disease-specific approaches for enhancing T cell accumulation in tumors of the CNS, further evaluation of VCAM-1^+^ expression across multiple tumor types is warranted. Nevertheless, our findings suggest that, for intracranial glioma, VLA-4^Hi^ lymphocytes may be able to encounter VCAM-1 and accumulate in tumor.

To better reflect the capacity for clinical translation, we confirmed that IL-7 exposure upregulated VLA-4 expression on hPBMCs from both healthy donors and patients with glioblastoma. While we observed reduced yields and less CD8^+^ T cell skewing when expanding hPBMCs with IL-7, we did find that IL-7–cultured hCD8^+^ T cells exhibited similar in vitro functionality to those being IL-2 cultured. Further optimization could include sorting infiltrative (VLA-4^Hi^) CD8^+^ T cells preadministration. This might also permit administering a reduced dose to patients, or one with more efficient tumor uptake. In our study, the percentage of the administered ALT dose that entered the brain was small, in keeping with other studies evaluating IV CAR-T therapy against murine CNS malignancy models (< 1%, lymphoma) (60). Though our approach did not induce overt toxicity/demyelination in brain or spinal cord when combining IL-7 ALT with αPD-1, we did observe a few scattered foci of hepatic cellular apoptosis/necrosis associated with lymphocytic infiltrate. An optimized VLA-4^Hi^ population could reduce the ALT dose required for efficacy and limit non-CNS organ infiltration.

A key limitation of our study is that we only evaluated the cytokines IL-2 and IL-7, as we have previously established that they can independently support T cell expansion (22). Similarly, we used doses of IL-2 and IL-7 that are known to expand T cells from both mice and humans. However, cytokines such as IL-15, IL-18, IL-21, and others have been used in varying doses to supplement lymphocyte expansion (61). We also note that the IL-7 receptor is downregulated after sustained exposure, and intermittent cycling with IL-7 may enhance the cytokine’s effects (62). Our study is confined to evaluating these two cytokines at the specified concentrations and exposure schedules. However, given the vast number of possible permutations, determining the optimal combination, concentration, and timing for cytokine supplementation is beyond the scope of this manuscript and may require a continuous process-development effort.

In conclusion, we find that expansion of autologous T lymphocytes with IL-7 enhances the ability of CD8^+^ T cells to accumulate within intracranial glioma, even in the setting of tumor-imposed T cell sequestration. Combination IL-7 ALT and T cell–centric immunotherapies increase survival in multiple models of glioma. Analysis of migratory integrins on lymphocytes finds that IL-7 increases CD8^+^ T cell expression of VLA-4. RNA-seq analysis finds that IL-7 increases transcription of genes associated with increased migratory integrin expression (*CD9*) as well as *S1PR1*, a G protein–coupled receptor whose stable expression on lymphocytes protects against their sequestration. These findings will be used to inform future clinical trials, where ALT pretreatment will be combined with T cell–activating therapies targeting glioblastoma (e.g., BRiTE, NCT04903795).

## Methods

### Sex as a biological variable.

For preclinical in vivo studies, we exclusively used females, as CT-2A was established in female mice (63) and superior engraftment of human hematopoietic stem cells has been observed in female NOD-scid γ (NSG) mice (64). Evaluation of clinical samples from volunteers and glioblastoma patients used hPBMCs from males and females (Table 1). Further demographic information in terms of reporting race and ethnicity were based on classifications within the electronic medical records system where available and categorized according to NIH policy (notice number: NOT-OD-15-089).

### Mice.

All mouse strains used, and housing conditions are described within the Supplemental Methods.

### Cell lines.

C57BL/6J syngeneic CT2A was originally provided by Robert L. Martuza (Massachusetts General Hospital, Boston, Massachusetts, USA). Generation of stably transfected sublines was performed in-house and used in previous studies (1). Similarly, U87MG was obtained from the American Type Culture Collection (ATCC, cat #HTB-14) and transfected in-house to stably express EGFRvIII. GL261 was obtained from the NCI (National Cancer Institute). Their generation and usage has also been described in prior studies by our group (2). All cell lines are authenticated and confirmed to be contaminant free via IDEXX Laboratories. CT2A, U87MG, and GL261 lines were maintained in culture using complete DMEM (Gibco, 10% FBS) and passaged using 0.05% Trypsin, EDTA (Gibco).

### Murine lymphocyte culturing.

Expansion processes for murine CD3^+^ cells are described within the Supplemental Methods.

### Leukapheresis and human PBMC culturing.

Human PBMC collection, processing and expansion are described within the Supplemental Methods.

### Tumor inoculation.

All tumor studies in this report placed tumors intracranially in mice and followed protocols described previously (65), which are described within the Supplemental Methods.

### In vivo adoptive lymphocyte transfer and antibody administration.

For adoptive lymphocyte transfer in this study, cells that had been expanded, as described in the murine lymphocyte culturing section, were administered as described within the Supplemental Methods.

### Tissue processing and flow cytometry.

Procedures for tissue processing and flow cytometry are based on our previously published protocols (4) and are described within the Supplemental Methods.

### Cytotoxicity.

Tumor cells were labeled with a viability dye before coculture. The process is described within the Supplemental Methods.

### Immune functional assays.

Immune functional assays were performed with flow-sorted human CD8^+^ T cells and are described within the Supplemental Methods.

### IHC & toxicity.

Toxicity studies were conducted in C57/Bl6 mice. Processing procedures are described within the Supplemental Methods.

### RNA-seq assays and analysis.

Flow-sorted CD8^+^ T cells were snap frozen in cell pellets and analyzed via bulk RNA-seq at Azenta life sciences. Analysis is described within the Supplemental Methods. PCA analysis was generated using BioJupies (66). Hierarchical cluster heatmaps were generated via the same method and using clustergrammer (Ma’ayan lab) (67). Gene Ontology (GO) biological process analysis was performed using the NIH database for annotation, visualization, and integrated discovery dashboard (DAVID) (53). Volcano plots were generated based on tests for differential expression (Wald test used to generate *P*-values and log_2_ fold changes) created with DESeq2 (68) and visualized using VolcaNoseR (69).

### Graphical illustrations.

The graphical abstract and experimental outlines in Figures 1–3, 5, 6, and Supplemental Figures 1, 3, and 7 were created with BioRender.com and exported under a paid license.

### Statistics.

Experimental results are presented as mean ± SEM unless otherwise stated. Statistical tests for all studies were completed using GraphPad v.10.3.1 (Prism). For comparisons in a single graph, 2-tailed Student’s *t* test or nonparametric Mann-Whitney *U* test was used, and for multiple comparisons, ANOVA (1-way or 2-way) with Holm–Šidák correction for multiple comparisons. Asterisks represent the significance level of any difference (*P < 0.05, **P ≤ 0.01, ***P < 0.001, ****P < 0.0001, *P* > 0.05 not significant). Sample sizes were selected due to practical considerations, with no formal power calculations performed. Efficacy studies following animals for survival were assessed for significance using a log-rank (Mantel-Cox) χ^2^ test. Independent study results were pooled if the effect of replication did not cause significant variation as assessed by 2-way ANOVA. All animals were randomized within genotype prior to treatment following tumor implantation. Survival was monitored with the assistance of technicians from the Duke Division of Laboratory Animal Resources (DLAR) who were blinded to study groups and followed animals to endpoint.

### Study approval.

All animal experiments were performed under Duke University IACUC experimental protocol (ID: A163-21-08). Stored anonymized human samples used were collected from glioblastoma patients or volunteers who provided written consent to undergo leukapheresis for T cell expansion for research (glioblastoma donors: Duke IRB #Pro00069444; Duke IRB #Pro00083828; healthy donors: Duke IRB #Pro00009403; approval for secondary analysis: Duke IRB #Pro00117663).

### Data availability.

Values for underlying data for all figures are available in the Supporting Data Values file. RNA-seq data (processed TPM counts for individual anonymized samples) have been deposited on the NCBI Gene Expression Omnibus (GEO) repository under accession number GSE288436. Institutional certification for deposition of genomic summary results was granted by Duke’s IRB review board.

## Author contributions

KS with assistance from KMH, SLC, PN, YZ, EMM, COR, EEB, BJP, SEW, PKN, GEA, BHS, KA, JHS, MK, and PEF designed, acquired data for, analyzed data, and wrote the manuscript for this study. JHS, MK, and PEF were responsible for overall supervision throughout the study.

## Supplementary Material

Supplemental data

Supporting data values

## Figures and Tables

**Table 1:**
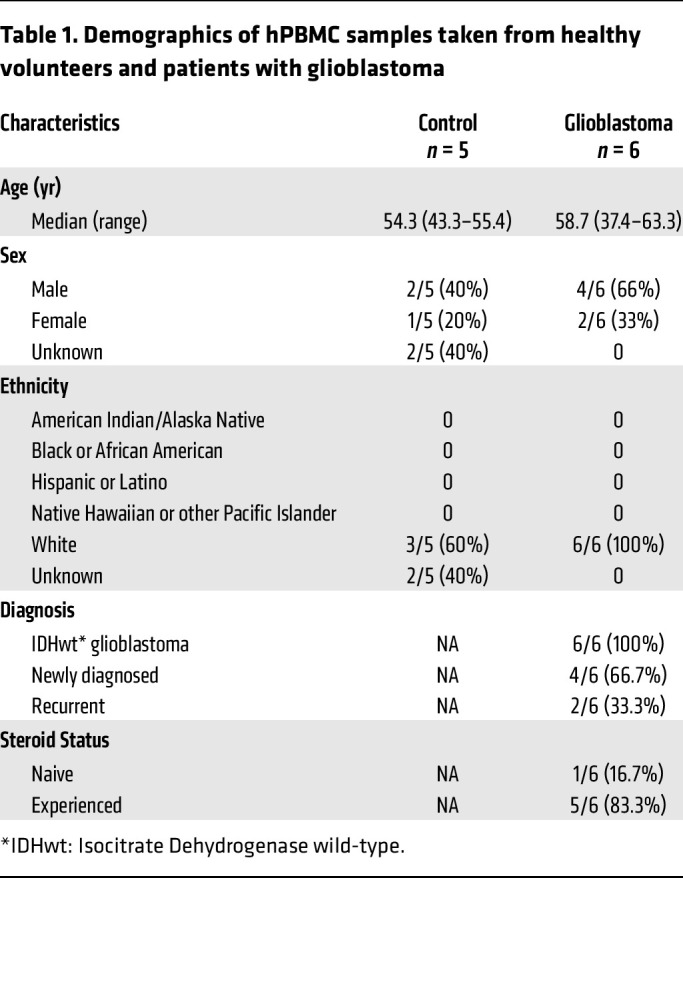
Demographics of hPBMC samples taken from healthy volunteers and patients with glioblastoma

## Version 1 04/17/2025

In-Press Preview

## Version 2 06/16/2025

Electronic publication
